# Green Tea Leaves Extract: Microencapsulation, Physicochemical and Storage Stability Study

**DOI:** 10.3390/molecules21080940

**Published:** 2016-07-26

**Authors:** James A. Zokti, Badlishah Sham Baharin, Abdulkarim Sabo Mohammed, Faridah Abas

**Affiliations:** 1 Department of Food Technology, Faculty of Food Science and Technology, University Putra, Malaysia UPM, Serdang 43400, Selangor, Malaysia; zoktii2002@yahoo.com; 2 Department of Food Science, Faculty of Food Science and Technology, University Putra, Malaysia UPM, Serdang 43400, Selangor, Malaysia; karimsabo@upm.edu.my (A.S.M.); faridah_abas@upm.edu.my (F.A.)

**Keywords:** green tea extracts, catechin, encapsulation, storage stability, shelf-life study

## Abstract

Green tea polyphenols have been reported to possess many biological properties. Despite the many potential benefits of green tea extracts, their sensitivity to high temperature, pH and oxygen is a major disadvantage hindering their effective utilization in the food industry. Green tea leaves from the Cameron Highlands Malaysia were extracted using supercritical fluid extraction (SFE). To improve the stability, green tea extracts were encapsulated by spray-drying using different carrier materials including maltodextrin (MD), gum arabic (GA) and chitosan (CTS) and their combinations at different ratios. Encapsulation efficiency, total phenolic content and antioxidant capacity were determined and were found to be in the range of 71.41%–88.04%, 19.32–24.90 (g GAE/100 g), and 29.52%–38.05% respectively. Further analysis of moisture content, water activity, hygroscopicity, bulk density and mean particles size distribution of the microparticles were carried out and the results ranged from; 2.31%–5.11%, 0.28–0.36, 3.22%–4.71%, 0.22–0.28 g/cm^3^ and 40.43–225.64 µm respectively. The ability of the microparticles to swell in simulated gastric fluid (SGF) and simulated intestinal fluid (SIF) was determined as 142.00%–188.63% and 207.55%–231.77%, respectively. Release of catechin polyphenol from microparticles in SIF was higher comparable to that of SGF. Storage stability of encapsulated catechin extracts under different temperature conditions was remarkably improved compared to non-encapsulated extract powder. This study showed that total catechin, total phenolic content (TPC) and antioxidant activity did not decrease significantly (*p* ≥ 0.05) under 4 °C storage conditions. The half-life study results were in the range of 35–60, 34–65 and 231–288 weeks at storage temperatures of 40 °C, 25 °C and 4 °C respectively, therefore, for improved shelf-life stability we recommend that microparticles should be stored at temperatures below 25 °C.

## 1. Introduction

Tea is an infusion of *Camellia sinensis* (tea plant) leaves. It is only second to water in terms of global consumption. Green tea is a product obtained from less fermented leaves of *C. sinensis*. Green tea has been reported to possess better health benefits compared to black and oolong teas which are more fermented. The main components of tea leaves that confer health benefits to green tea are its polyphenolic compounds, including epicatechin (EC), and epicatechin gallate (ECC), epigallocatechin (EGC), and epigallocatechin gallate (EGCG). Epidemiological data have shown that consumption of tea has an inverse relationship with risk of certain chronic and degenerative diseases including certain forms of cancers, cardiovascular diseases, neurodegenerative diseases, diabetes, obesity, Alzheimer’s disease, weight loss, etc. [1,2]. It is also reported that catechins from green tea exhibit diverse biological properties including antioxidant, anti-carcinogenic, and anti-inflammatory activities [3]. Tea is listed as a functional food because of the health promoting characteristics. Functional foods play preventive roles because of their ability to reduce factors that compromise good health [4]. Green tea catechins have been incorporated into various foods, including breads, cakes, biscuits, yoghurts, meats and pastries [5,6]. The therapeutic value of green tea combined with is popularity presents an opportunity to positively affect the outcome of risks of disease globally.

However, green tea catechin polyphenols have been reported to undergo degradation/epimerization when exposed to the effects of temperature, pH, light, oxygen and enzymatic action, which all reduce the efficacy of the polyphenols [7,8]. The bitterness and stringency of green tea has been attributed to the presence of catechins [9]. Many consumers do not like to take green tea because of this [9].

Furthermore, studies have shown that green tea extracts after drying are hygroscopic and sticky because of the presence of some sugars and therefore are not shelf-stable and can absorb moisture and degrade very easily [10]. The stickiness has been attributed to the low glass transition temperatures of glucose, fructose and citric acid [11]. It has been suggested that it is important to add high molecular weight sugar compounds with high glass transition temperature [12] in order to solve some of the problems associated with instability of the products. The effects of pH, temperature and other ingredients of soft drinks on the stability of brewed green tea catechins has been widely reported in [7,13,14]. This may be the reason why functional food developers have been looking for ways to curtail the degradation and also ensure that the stability, bioactivity and bioavailability of tea catechins are guaranteed during processing, storage and consumption. Food products must not only be seen to provide nutritionally significant dose of the bioactive compounds, but remain functional as long as required [15,16,17]. Encapsulation has been seen as the best solution to all these challenges associated with green tea catechin extracts [18,19,20,21].

Stability of a food powder is defined as a state where its physical, chemical and microbial characteristics are maintained without change during handling throughout the storage period. Examples of physical properties of powders are particle size, shape, porosity, size diameter, density, solubility, and hygroscopicity and surface characteristics. The chemical characteristics of food powders are related to their composition, as well as their interactions of other components with the structure of the food [22].

As a strong antioxidant the stability of catechin is a very important factor to be considered if it is to be used as a functional food ingredient. Spray drying has been used for microencapsulation by formulators of functional food ingredients to turn liquids into easy to handle powders, protect their stability and bring about sustained release of polyphenols [23,24,25]. More importantly, spray drying technology has been widely exploited by food industries because it is an already established technology, it has the ability to produce large amount of microcapsules, many approved shell materials for different uses exist, one can produce different varieties of particle sizes and is can be used for heat sensitive food ingredients [25,26]. During encapsulation sensitive bioactive compounds are packaged within the carrier material which protects the sensitive core materials against adverse condition of the environment and controls the release of the bioactive compounds.

Maltodextrin is one of the wall materials that is commonly used in the industry for encapsulation of functional food ingredients because of its unique characteristics [22,27] Gum arabic is a coating material derived from two species of acacia tree, *Acacia seyal* and *Acacia senegal*. Gum arabic is primarily used as a stabilizer in food industry. It is a good emulsifier with film-forming properties and low viscosity in aqueous solution which helps spray-drying [28,29]. Gum arabic is completely edible and resistant to a number of physicochemical conditions such as those encountered in gastrointestinal fluid, which makes it suitable for encapsulation of green tea catechins [30]. Chitosan is a non-toxic biopolymer that is biodegradable and biocompatible with much potential for use in biotechnological applications. Because of its mucoadhesive, cationic and reactive functional groups; chitosan is used in controlled released formulations in the pharmaceutical industry [31,32]. Chitosan has proved to be valuable in the wine and juice industry too. Recently polyphenolic extracts from olive leaves were encapsulated by spray drying using a chitosan coating with high loading efficiency compared to particles made by extrusion [32,33]. In this study catechin is expected to serve as the main health promoting agent, however, because chitosan has shown some beneficial health properties its inclusion as a carrier material will be of relevance too. Encapsulation of catechin extracts using maltodextrin and gum Arabic along with chitosan is a novel approach. Catechin extracts from green tea leaves are currently available in the market in their pure forms, but they are very expensive and may not be economical enough for use as functional food ingredients unlike in the pharmaceutical industry.

The aim of this study was to produce low cost catechin extracts as alternatives to pure forms by encapsulating cathechins in moderately inexpensive polymer materials which are shelf-stable and affordable by low cost functional food manufacturers, and to evaluate the stability at different storage temperatures after microencapsulation.

## 2. Results and Discussion

### 2.1. Physicochemical Properties of Green Tea Catechin Microparticles

The effect of carrier materials (maltodextrin, gum arabic and chitosan) on the moisture, water activity, hygroscopicity, particles size distribution and morphology, stability and the efficiency of the entrapped core material were determined in this study.

#### 2.1.1. Moisture Content

The analysis of variance and mean experimental results of physicochemical properties of green tea extract powder produced under different conditions using different wall materials are listed in Table 1. The moisture content was generally low (4.78% ± 0.04%–2.31% ± 0.01%), but microparticles with high hydrocolloid content were sticky and gummy. There was a significant (*p* ≤ 0.05) difference in the moisture content of encapsulated green tea extract and that of the free green tea powder (control). The results showed that microencapsulation influenced the moisture content significantly (*p* ˂ 0.05). The change in the moisture content of the microparticles depends significantly (*p* ˂ 0.05) on the concentration and the type of wall material used. The microparticles with higher or double hydrocolloids had higher moisture content. It was observed that with higher hydrocolloid levels, the total solids decrease and that might have been responsible for the higher moisture contents. It has been reported that when the concentration of carrier material increases moisture content decreases due to the increase in total solids [34]. This result is in agreement with the reports of other researchers [35,36,37]. The interactions between the wall materials and the core material had a significant effect on the moisture content of the samples. It has been suggested that for better stability the moisture content of instant tea powder should be between 3% and 5% [38].

#### 2.1.2. The Water Activity

Water activity (a^w^) is a very important parameter for spray-dried food powder, because it has great influence on the stability and shelf-life of the microparticles produced. When the water activity is high it is an indication that there is available free water for biochemical activity to take place within the system, therefore shelf-life is shorter. The a^w^ of the microencapsulated green tea extract was between 0.36 and 0.28 and that of the free green tea extract (control) was 0.45 (Table 1). Similarly, as the concentration of hydrocolloids increased the water activity of the microparticles decreased. This can be attributed to the binding capacity of the hydrocolloids. Maltodextrin and gum arabic are moisture binders. There was a significant difference (*p* ˂ 0.05) among the treatment groups. Moisture contents were generally low. To provide better stability it has been suggested that a water activity of the microparticles should be lower than that of the free powder [39].

#### 2.1.3. Hygroscopicity

The hygroscopicity test results of the microparticles is presented in Table 1. A significant difference (*p* ˂ 0.05) occurs among the various wall materials used. Free green tea extracts (control) were more hygroscopic, while the encapsulated green tea extract powder was less hygroscopic. This shows that the carrier materials acted as moisture binders thereby reducing the ability of the core material to absorb moisture. The blends of the various carrier materials showed better results as moisture binders compared to maltodextrin and gum arabic when used alone. The behaviour observed in this work has been reported in similar previous studies [40,41,42,43].

#### 2.1.4. Particle Size Distribution

**Figure 1:**
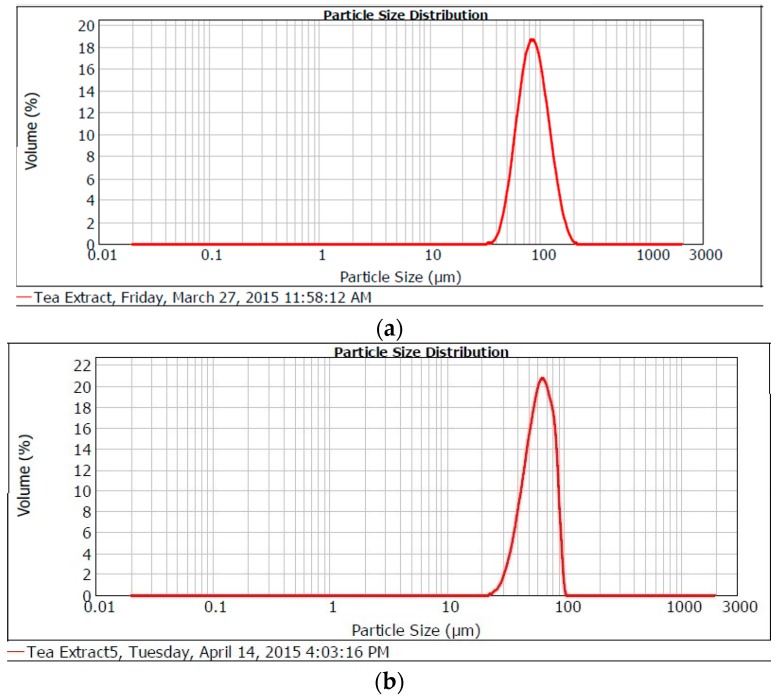

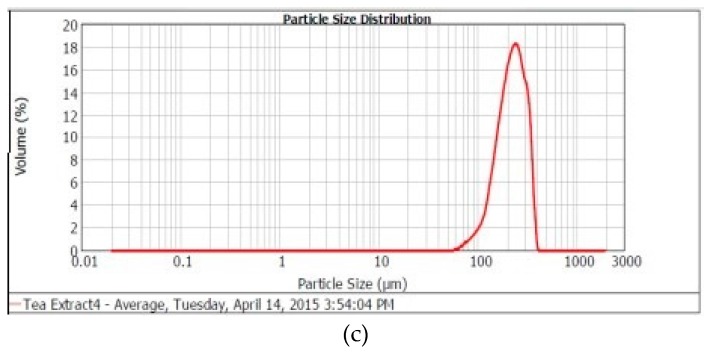
Particle size distribution curve of spray dried Microparticles (**a**) Placebo peak; (**b**) maltoxtrin-gum arabic peak (**c**) maltodextrin:gum arabic:chitosan loaded microparticles.

The spray dried particles size distribution is presented in Table 1. Particle size distribution showed single mode with a mean d(4,3) size population between 40.43 and 226.79 µm. The more homogenous the particle size distribution the more stable the core materials within the microcapsules. Figure 1a–c show particle size distribution curves of microparticles prepared from different wall materials (maltodextrin, gum rabic and chitosan), (a) placebo curve; (b) curve from maltodextrin and gum with core material (c) curve from maltodextrin, gum rabic and chitosan with core material. In conventional spray drying processes, most often the final particle sizes generated are in the range of 5 µm to 250 µm in diameter because of the differences in droplet size after dispersion [44,45]. When the droplet/particle sizes vary, the microparticles obtained from the spray drying process will show different residual activity and therefore the quality will vary too [37]. Since a 0.7 mm nozzle was used in our study, it was expected that microparticles of moderately larger sizes would be formed as observed.

### 2.2. Effects of Wall Materials on Encapsulation Efficiency (ME), Total Phenolic (TPC) Content and Antioxidant Activity (DPPH)

**Figure 2:**
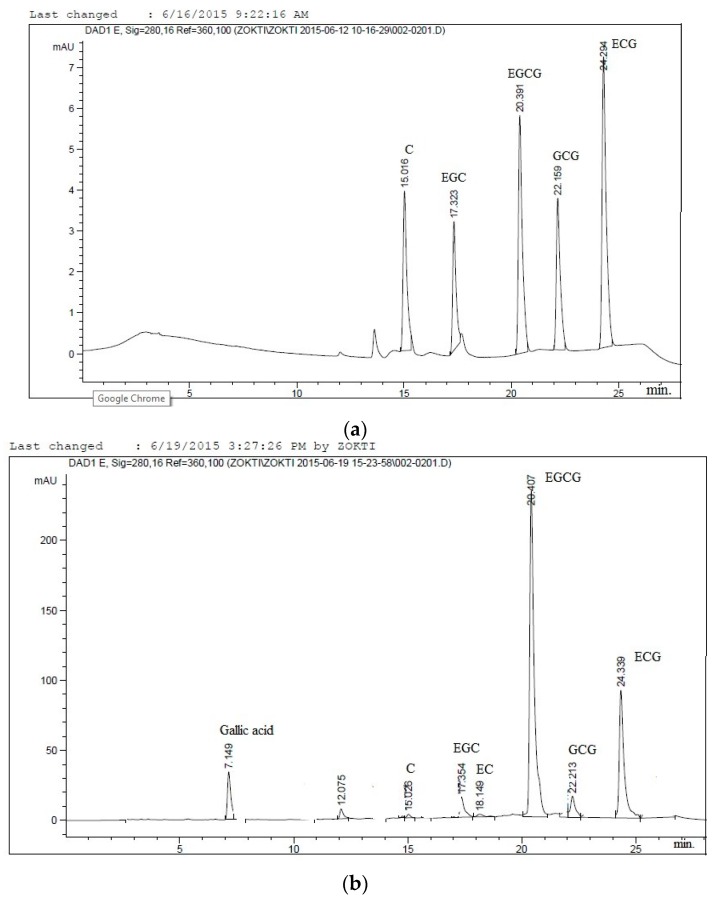
HPLC chromatograms (**a**) catechin standards; (**b**) chromatograms from in vitro studies.

Microencapsulation efficiency is determined with the sole aim of evaluating the loss of core material before, during and after processing microparticles. Microencapsulation efficiency is the percentage of entrapped catechin over the total catechin in the system. Factors that affect the retention of the core materials include spray drying conditions, composition and physical properties of the suspension before drying, and mass ratio of the active ingredient. It is expected that an ideal microencapsulation technique should result in minimum loss of the core material during spray drying [46]. The efficiency of microencapsulation was determined using an HPLC analysis method based on total catechins contents. Figure 2a,b represent HPLC chromatograms of a catechins standard and catechins identified in the green tea extracts.

**Figure 3:**
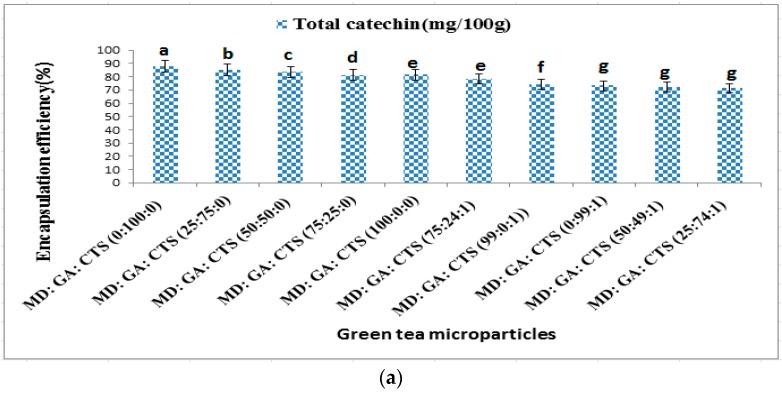

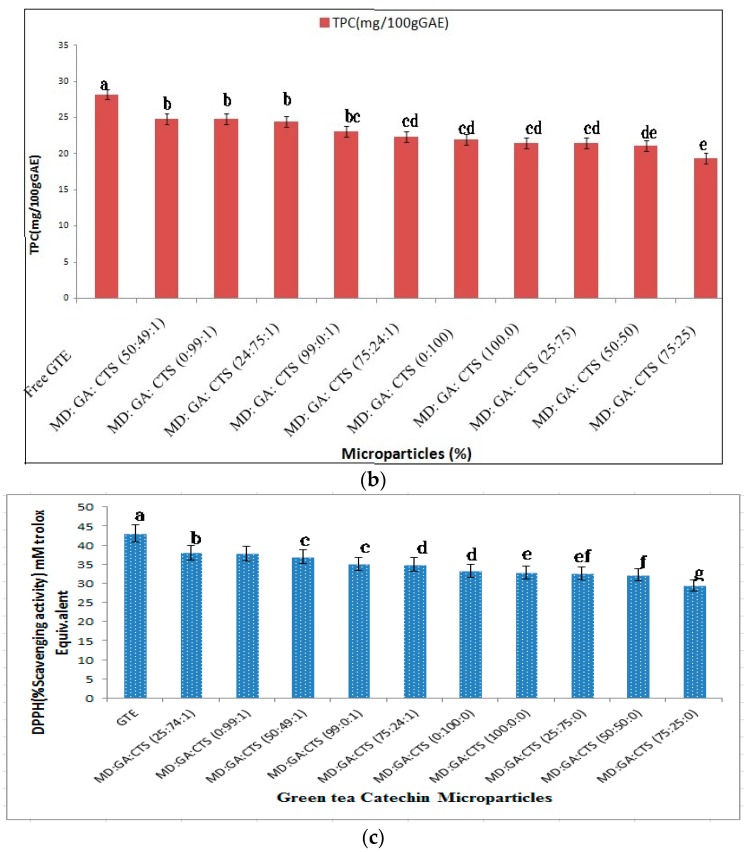
Effects of wall materials on (**a**) encapsulation efficiency of entrapped catechin; (**b**) total phenolic contents (TPC) of green tea microparticles; (**c**) DPPH radical scavenging activity of green tea microparticles. Values with different letters are significantly (*p* ≥ 0.05) different. The values are represented as the means ± SD. (MD—maltodextrin, GA—gum arabic, CTS—chitosan).

The mean value of three independent determinations of encapsulation sufficiency is presented in Figure 3a. The encapsulation efficiency was in the range of 88.04%–71.41%. The results showed that the type of wall material used affects the efficiency of encapsulation. The formulation with gum arabic 100% followed by blends containing higher grabicbic ratios and maltodextrin were significantly (*p* ≥ 0.05) more efficient in encapsulating the catechin bioactive compounds. Formulations containing chitosan did not differ significantly (*p* ≥ 0.05) from each other. The encapsulation efficiency was generally fairly good (71.41%–88.04%); considering the viscosity of the feed suspension and low amount of core materials (5%) used in preparing the liquid feeds knowing that the highest and the smallest particles are lost with the exhaust of the spray dryer. The low encapsulation efficiency experienced from the formulations containing chitosan may be as a result of the high viscosity of chitosan. It is reported that viscous liquid feeds are not suitable for spray drying and are not able to form well-coated microparticles [26]. During the preliminary studies it was found that using chitosan more than 1% (*w*/*v*) concentration could not produce well-coated microparticles because it was very viscous for spray drying, affecting the rate of formation of droplets suitable for atomization during the spray drying process. Spray drying temperature was also an important factor because it was found that it affected the total catechins significantly. When the temperature was above 150 °C it affected the total catechins concentration, whereas temperatures below 150 °C could not produce good powders (results not shown).

Figure 3b,c present the results of total phenolic content (TPC) and percentage DPPH scavenging capacity of encapsulated green tea microparticles, respectively. The total phenolic contents (TPC) of the various formulations as determined using Folin–Ciocalteu colorimetric method were in the range of (24.90 ± 0.05–19.32 ± 0.14 (g/100 g GAE). MD:GA:CTS (25:74:1) blend had the highest TPC content followed by MD:GA:CTS (99:1:0) and MD:GA:CTS (50:49:1) blends. Previous studies on total phenolic content in green tea showed higher total polyphenolic contents [47]. The DPPH scavenging capacity was between 29.5% ± 0.34%–38.0% ± 1.27% (Figure 3c). The formulation with 25:74:1 (maltodextrin:gum arabic:chitosan) showed the highest antioxidant effect. Interestingly, it was noted that the formulation that produced the highest total polyphenolic compounds (24.74 g/100 g GAE) produced the lowest catechin encapsulation efficiency (71.99%). This can be attributed to the combined antioxidant effects of gum arabic, chitosan and green tea catechins. The trend was generally observed in this study. The antoxidative effect of chitosan-containing microparticles has been reported in previous similar studies [32].

### 2.3. Swelling Studies of Microparticles

**Figure 4:**
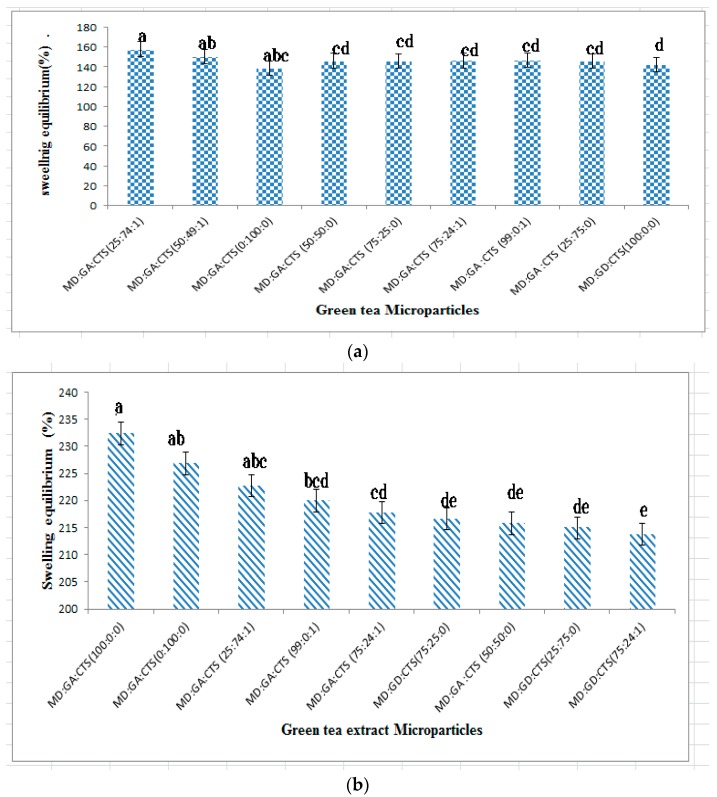
Swelling potentials of microparticles in (**a**)simulated gastric fluid (pH 2.3) and (**b**) simulated intestinal fluid (pH 7.4) at 37 °C for 120 min. Values with different letters are significantly (*p* ≥ 0.05) different. Values are presented as mean ± SD. (MD—maltodextrin, GA—gum arabic, CTS—chitosan).

Characterization of swelling of microparticles was carried out. It was anticipated that the release of microparticles may occur by swelling and degradation. The extent of microparticles swelling depends on the medium in which microparticles are immersed, as well as their size. The microparticle swelling was evaluated gravimetrically. Since the particles were prepared as food ingredients, release of catechin was characterized under conditions mimicking digestion under both gastric pH (2.3) and intestinal pH (7.4) conditions. The capacity of the microparticles to swell under these conditions was evaluated. The microparticles of green tea from the formulated wall materials demonstrated swelling ability of between 142.00% and 188.65% when incorporated into the simulated gastric fluid (SGF) over a period of 120 min (Figure 4a); whereas the swelling index in simulated intestinal (SIF) was between 207.55% and 231.77% (Figure 4b). There was a significant difference (*p* ˂ 0.05) in the swelling index of microcapsules containing chitosan in SGF and SIF when compared to others without chitosan blends. Swelling of microparticles containing a high ratio of maltodextrin reached equilibrium within the first 40 min, whereas blends with maltodextrin rabicarabic/chitosan reached swelling equilibrium within 60–100 min. The pH of the medium played a significant (*p* ˂ 0.05) role in the swelling equilibrium of the catechin-loaded microparticles. All the microparticles swell less in the SGF except those containing chitosan blends compared to those incubated in SIF conditions. There are very limited reports on pH dependent swelling index and stability of catechin microparticles in related studies [10,32]. Reports on insulin pH-dependent swelling and chitosan nanoparticles shows that chitosan swells more at the gastric pH, compared to the intestinal pH [31,32]. In this study the swelling pattern of the microparticles can be attributed to the high swelling of chitosan and its ability to uncoil the carbohydrate molecules to an extended structure with higher molecular weight because of the pH controlled electrostatic interaction between anions and chitosan films. There are few reports on microparticles that show pH-motivated swelling, but it is assumed that the mechanism of pH-sensitive swelling may be as a result of protonation of the amino group of chitosan when the pH decreases [48]. The microparticles formulated with MD:GA:CTS (25:74:1) showed the highest rate of swelling in simulated gastric fluid (SGF), while 100 rabicarabic showed less swelling. Conversely rabicarabic and maltodextrin 100% exhibited higher swelling capacity in simulated intestinal fluid (SIF). Higher swelling of chitosan nanoparticles in acidic pH media compared to intestinal pH has been observed [32]. Other factors responsible for the observed degree of swelling may have been particle size and catechin-wall material interactions.

### 2.4. Release of Phenolic Compounds (TPC), DPPH Scavenging Activity and Total Catechin (TC) from Simulated Gastrointestinal Fluids (pH 2.3)

**Figure 5:**
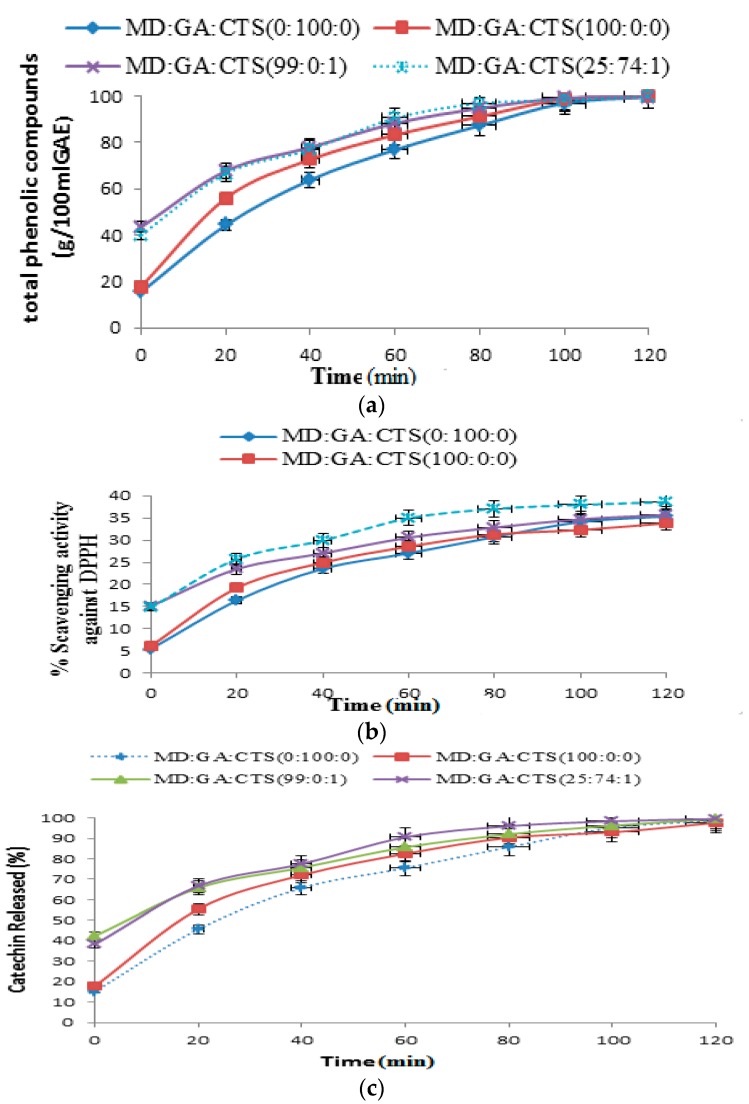
In vitro release profiles of (**a**) total phenolic content (TPC). (**b**) In vitro release DPPH scavenging activity; (**c**) In vitro release profiles of total catechins (TC) from microparticles in simulated gastric fluid (pH 2.3) at 37 °C for 120 min. The values are represented as the means ± SD. (MD—maltodextrin, GA—gum arabic, CTS—chitosan).

**Figure 6:**
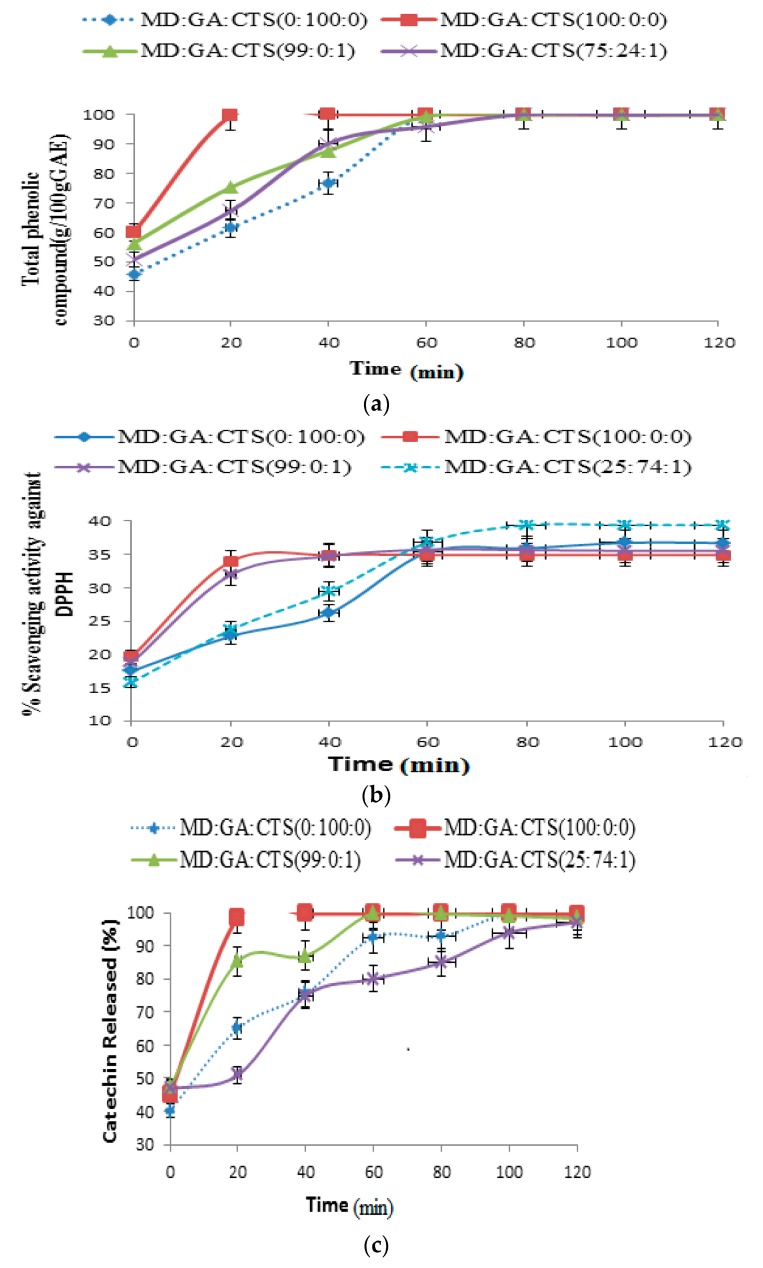
In vitro release profiles (**a**) total phenolic content (TPC); (**b**) DPPH scavenging activity; (**c**) TC from microparticles in simulated intestinal fluid (pH 7.4) at 37 °C for 120 min. The values are represented as the means ± SD. (MD—maltodextrin, GA—gum arabic, CTS—chitosan).

Highly reactive free radicals are considered harmful to human health. Therefore it is important to determine the stability of the constructed green tea microparticles under conditions that are similar to those of the gastrointestinal tract. Previous studies have shown that the main phenolic compound in green tea is catechin [49,50,51].Therefore identification and quantification of catechin polyphenol fractions as reference phenolic compounds were undertaken in this study. Retention of bioactive compounds from the in vitro study was evaluated based on the release of total phenolic contents (TPC), antioxidant scavenging capacity (DPPH) and total catechins (TC) dissolved under simulated gastric fluid (SGF) and simulated intestinal fluid (SIF) conditions. Catechins are highly soluble in alkaline fluids such as the one produced in the human gastrointestinal region. Low bioavailability (˂2%) has been associated with the degradation of catechin under intestinal pH conditions [8,52,53]. In order to obtain information on the catechin compounds from the microcapsules during in vitro studies; spectrophometric analysis and high performance liquid chromatography (HPLC) were used to quantify TPC, DPPH and TC retention in the simulated gastrointestinal fluid (SGF and SIF). The result of the in vitro release profile of polyphenolic contents from microparticle powder at gastric pH (2.3) and intestinal pH (7.4) using Visking tubing dialysis bags are reported in Figure 5a–c and Figure 6a–c.

Figure 5a shows the release pattern of TPC in SGF (pH 2.3). The release of total phenolic contents from microparticles prepared from 100% maltodextrin an rabicarabic was 13.6% ± 0.47% during the first 20 min and increased to 51.36% at 60 min incubation period and then they gradually emptied their contents by the end (120 min). For maltodextrin rabicarabic/chitosan microcapsules (MD:GA:CTS (25:74:1), MD:GA:CTS (99:0:1) blends; it was observed that microparticles released more than 50% of the total polyphenols in the first 20 min before gradually reaching a plateau at the end of 120 min. The change in the antioxidant scavenging activity (DPPH) was similar to that of TPC (Figure 5b).The release of phenolic compounds from the microparticles is a result of dissociation of microparticles due to the change in pH of the medium [54]. The release of TPC and DPPH during the incubation period showed that at the gastric intestinal fluid condition the release of total phenolic contents and antioxidant scavenging activity reached a maximum value at 120 min. The lowest release was observed irabicarabic and maltodextrin 100% formulations. The releases from blends of maltodextrin rabicarabic and chitosan were faster than for maltodextrin an rabicarabic at pH 2.3. This report shows that MD:GA:CST (25:74:1) exhibited the highest release rate, while MD 100% release was the least under simulated gastric fluid conditions (pH 2.3).

Figure 5c presents the release pattern of TC under SGF conditions. The total catechins were obtained as the sum of the five main catechin compounds (EC, EGC, EGCG, GCG and ECG). When the release pattern of TC was evaluated under gastric fluid conditions, we found out that 15.2% to 42.29% of total catechins were released within the first 20 min and 82.45% to 90.55% of the contents were released at the end of 60 min. The microparticles gradually released their content at the end of 120 min, except for 100% maltodextrin microparticles which release their content before the end of the incubation period (120 min). However, it was observed that microparticles prepared with maltodextrin and gum arabic 100% did not show a specific pattern of release compared to microparticles that were prepared with chitosan, which showed faster release under SGS conditions.

### 2.5. Release of Phenolic Compounds (TPC), DPPH Scavenging Activity and Total Catechin (TC) from Simulated Intestinal Fluids (pH 7.4)

Figure 6a–c show the release of microparticles in simulated intestinal fluid (SIF). The microparticles showed higher rates of TPC, DPPH and TC release than those of simulated gastric fluid (SGF). All the microparticles released over 82%–95% of their contents between 20 and 60 min; except for microparticles containing maltodextrin rabicarabic:chitosan (25:74:1) blend an rabicarabic 100%. When the release of catechin phenolic compounds in SGF and SIF was taken into consideration, we found out that microparticles provide higher release under intestinal fluid conditions compared to gastric conditions. Therefore total phenolic compound levels are positively associated with phenolic substances in the green tea samples. This pattern of release can be attributed to microparticles—water interactions because of the amorphous physical condition of the powders which results in enhanced solubility. A similar result has been reported in previous study [26]. In this study, the high solubility of maltodextrin and the hydrophilic nature o rabicarabic may be the responsible for the rapid release of catechin extracts observed under the simulated gastrointestinal fluid conditions. The delay in the release of the particles containing chitosan may be associated to its high molecular weight and low solubility in water when compared to that of maltodextrin an rabicarabic. This behaviour has also been reported previously [55,56]. In another study designed to determine the release of bayberry polyphenols [57] less release was observed under SGF conditions, while most of the release (82%–98%) took place during the first 40 min under SIF conditions. The results of in vitro retention of catechins showed that the rate of retention of catechin in the microparticles was in the order orabicarabic > maltodextrin rabicarabic > maltodextrin > maltodextrin rabicarabic-chitosan. The carrier blends of maltodextrin:gum Arabic:chitosan (25:74:1) showed the lowest catechin retention, while 100 rabicarabic carrier agent gave the highest retention capacity. The HPLC analysis of catechin during the in vitro study did not reveal the presence of additional peaks (ghost peaks) after the period of incubation in the simulated gastrointestinal fluids. This was an indication that the catechin compounds did not undergo any changes (epimerization, oxidation, metabolization or degradation) in the simulated gastrointestinal fluids during the period of incubation, and are likely to remain in their active forms ready to be absorbed after consumption. The results show that the sustained release of green tea catechins from the microparticles can lead to a reduction of chemical degradation under gut conditions. Thus, we could exploit the ability of the microparticles to stabilize the green tea catechins for food applications to deliver catechin in drinks. Several studies using animal and human subjects have demonstrated the fate of catechin in the intestines after consumption [53,58,59]. The results of the in vitro test in this study indicate that microparticles adequately protected catechin polyphenols and they may be available/stable for absorption in the intestine.

### 2.6. Shelf-Life/Stability Study of Green Tea Extract Microparticles

**Figure 7:**
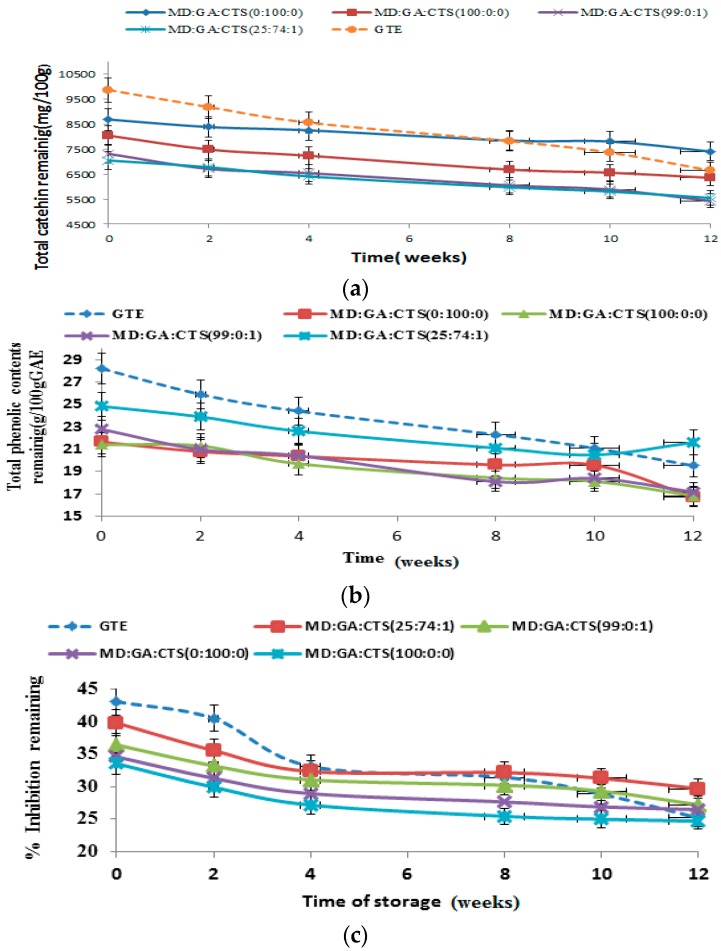
Storage study of green tea microparticles powder at 40 °C for 12 weeks showing (**a**) stability of total catechin in powder; (**b**) total phenolic content (TPC); (**c**) and antioxidant activity of microparticles. All values are means of three replicate sample determinations ± SD. (MD—maltodextrin, GA—gum arabic, CTS—chitosan).

**Figure 8:**
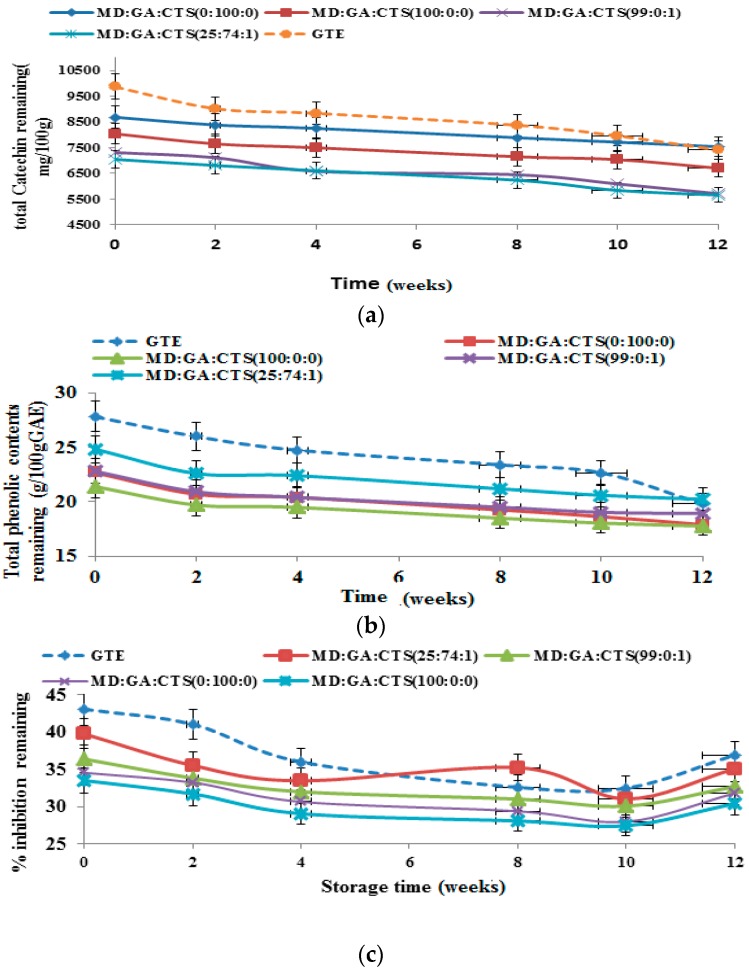
Storage study of green tea microparticles powder at 25 °C for12 weeks showing (**a**) total catechins; (**b**) total phenolic content; and (**c**) antioxidant activity stored at 25 °C for 12 weeks. Values are means of three replicate sample determinations ± SD. (MD—maltodextrin, GA—gum arabic, CTS—chitosan).

**Figure 9:**
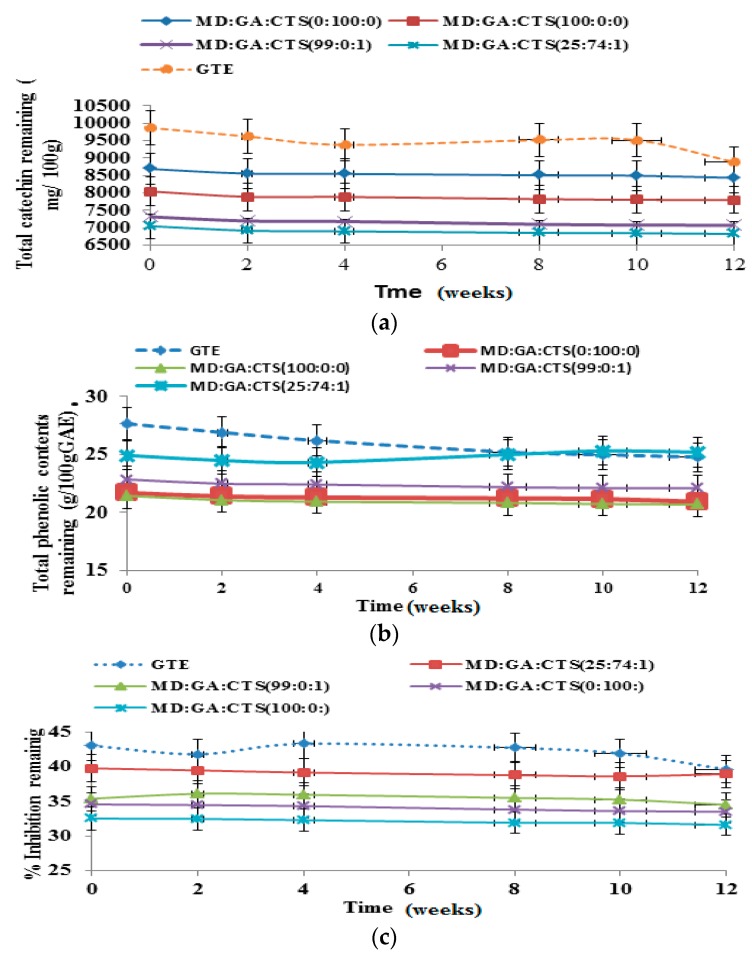
Storage study of green tea microparticles at 4 °C for 12 weeks showing (**a**) stability of total catechins content; (**b**) total phenolic content (TPC); and (**c**) antioxidant activity. All values are means of three replicate samples. (MD—maltodextrin, GA—gum arabic, CTS—chitosan).

The results of changes in total catechin (TC), total phenolic contents (TPC) and antioxidant activity (AOC) of the spray-dried green tea catechin microparticle powder and non-encapsulated (free) green tea catechin extract powder (control) during storage at different temperatures 40 °C, 25 °C and 4 °C) for 12 weeks are presented in Figure 7a–c, Figure 8a–c and Figure 9a–c, respectively. Figure 7a shows that at the end of storage at 40 °C for 12 weeks the percentage TC retained was between 67.38% and 81.82% with the control samples exhibiting the greatest degradation rate (32.62%); whereas 100 rabicarabic and 100% maltodextrin microcapsules showed the least degradation. The stability of blends of maltodextrin rabicarabic:chitosan (25:74:1) and maltodextrin:chitosan (99:1) was not significantly (*p* ≤ 0.05) different.

The TPC of the microparticles under the same storage conditions was in the range of 68.22%–83.32% (Figure 7b). It was observed that non-encapsulated catechin powder showed the least TPC, whereas 25:74:1 maltodextri rabicarabic:chitosan blend exhibited the highest TPC followed by 99:1 maltodextrin-chitosan blend rabicarabic (100%) retained about 80% of the total phenolic content.

Figure 7c presents the antioxidant capacity (AOC) evaluated using DPPH radical scavenging capacity under the same storage conditions (40 °C). The DPPH scavenging capacity of the retained compounds was in the range of 60.0%–82.87%. Free catechin extract powder exhibited the lowest % inhibition, followed by maltodextrin an rabicarabic microparticles.

Figure 8a is the result of the evaluation the stability of the TC, TPC and DPPH inhibition of green tea extract microparticle powder and non-encapsulated (free) green tea extract powder stored at 25 °C for 12 weeks. The value of TC retained was in the range of 74.15%–81.10%. Microparticles prepared with maltodextrin an rabicarabic (100%) exhibited the best catechin retention capacity, while the control showed the highest rate of degradation. The value of TPC retained at 25 °C was as presented in Figure 8b. The amount of TPC retained at the end of 12 weeks storage period was between 68.01% and 83.92%. There was no significant (*p* ≤ 0.05) difference in the rate of degradation of TPC in respect to (25:74:1), (99:0:1) and 100% maltodextrin except for 100 rabicarabic and free GTE powder. Free GTE powder samples exhibited the highest TPC degradation at 25 °C. The rate of DPPH scavenging capacity of the samples tested at 25 °C is shown in Figure 8c. It was observed that the percentage inhibition of DPPH scavenging capacity was in the range of 85.72%–90.84% at the end of the storage period. We found out that the microparticles including the control showed reduction in the rate of percentage inhibition in the 1st to the 8th weeks of storage and then increased marginally by the 12th week.

The changes in total catechin (TC), total phenol content (TPC) and antioxidant capacity (AOC) of spray dried powder at temperature of 4 °C are shown in Figure 9a–c. The TC, TPC and DPPH activity decreased in the range 2.43%–10.16%, 1.80–10.85 and 2.09%–8.01%, respectively. It was observed that the total catechin of the free crude extract decreased by over 10%; but there was no significant (*p* ≤ 0.05) difference in degradation rate of any of the encapsulated microparticles prepared from the different carrier materials. About 96% of the total catechin was retained at the end of 12 weeks storage time. It can be observed from Figure 9b that the total phenol contents in microparticles prepared with (25:74:1) increased by 1.9%, whereas over 95% TPC was retained from extracts prepared with (99:0:1), MD 100%, and GA 100% formulation. As for the effects of storage temperature (4°C) on the antioxidant activity of catechin microparticles, the (25:74:1) and (99:0:1) blends exhibited the best antioxidant activities at the end of the storage period. Previous studies have reported a decrease of polyphenols during stability studies at 8 °C and 25 °C [60,61]. Due to the presence of catechin compounds the storage stability of the spray-dried powders exhibited good antioxidant capacity at the end of 12 weeks storage at 4 °C and lower stability under 25 °C and 40 °C storage conditions. At the end of the storage time the catechin retention and antioxidant potentials of the spray dried microparticles were high irrespective of the wall materials used. The carrier materials did not show any significant (*p* ≥ 0.05) influence on the stability of catechin microparticles when stored with refrigeration (4 °C) compared to 25 °C and 40 °C.

### 2.7. Shelf-Life Testing

Studies on kinetic modelling of the stability were carried to determine the rate of chemical reaction during the storage period. First-order kinetics were used for the analysis. The data obtained were used to plot a scatter diagram of a semi-log of retention of all the analytes vs. time to determine the rate constant (k) reaction order for each of the green tea microparticle powders. For a reaction to be described as a first-order reaction; a plot of ln catechin retained against time should be linear (a straight line). Data plots of catechin retained versus time were linear. The *r*^2^ of all the samples were in the range of 0.88–0.99 which was close to 1 (Table 2), showing that the results fitted well a first-order model.

The half-life (t_1/2_) of the various green tea microparticles powder and the control was determined using the equation:

T_1/2_ = ln/k (1)

where k is the slope of the regression line which also referred to as the rate constant.

This is equivalent to:

T_1/2_ = 0.693147/k (2)

The value of k obtained from the regression equation was substituted to obtain the t_1/2_ for each catechin microcapsule type.

The results showed that reaction rate k increases with increased temperature. The half-life (t_1/2_), which is the time required for the catechin to reduce by 50% of its initial value was in the range of 35–60, 34–56 and 231–288 weeks at the storage temperatures of 40 °C, 25 °C and 4 °C, respectively. For comparison, it was reported [62] that for the degradation kinetics of beetroot pigment the reaction rate (k) and half–life was observed at a higher water activity of 0.64. Thus we can explain the degradation based on the fact that it is controlled by the mobility of the reactants, whereas in a similar report [63] it is shown that with an increase in water activity (a^w^) there will be increase in oxygen concentration which in turn accelerates the degradation rate because the presence of oxygen accelerates the reaction rate. When the a^w^ is low (dry environment) water is bound strongly to the surface polar sites and becomes available for reactions to take place. This explains why there is low reaction at low a^w^ in the microparticles compared to the free extract powder (non-encapsulated samples) as observed in this study. The water activity, moisture and bulk density of the green tea microparticles were generally low, as observed in Table 1.

There is a correlation in the result of the bulk density of the microparticles with the result of the kinetic study. When the bulk density is higher the surface area exposed becomes lesser. This may be one of the reasons for the higher degradation rate observed in the case of the control sample (non-encapsulated powder) with lower bulk density. Reference [27] reported that when there is less air, the powder becomes more occluded therefore there will be greater oxidation/degradation of the powder, which reduces the stability of the bioactives compounds during the storage period.

In this study it was found that temperature influenced the stability of the studied parameters (TC, TPC, DPPH) of the crude extract and the encapsulated powders. The results show that catechin polyphenols were more stable at lower storage temperature. To reduce the degradation of catechin it is recommended that catechin microparticles should be stored at temperatures below 25 °C or 40 °C. This result is consistent with the findings of [28,51,64,65] on plant polyphenol storage stability studies. However, this study showed that catechin microparticles were relatively unstable in MD:GA:CTS (25:74:1) and (99:0:1) blends, than in 100% maltodextrin and 100% gum arabic. Although the blends of carrier materials containing chitosan showed less protection of catechins the study revealed higher TPC and DPPH scavenging activity than in formulations without chitosan. The reason for greater degradation of catechin at higher storage temperatures may be due to chemical decomposition as a result of oxidation, hydrolysis or epimerization which has been suggested by previous researchers [7,13,53,66,67]. The slight increase in the values of total phenolic content and antioxidant activity observed at at the end of the storage period at higher storage temperature may be attributed to the reactions of phenols with the protein fraction of chitosan or gum arabic forming complexes, resulting in higher readings. In a study on maltodextrin/pectin microparticles for nutraceutical extracts produced by spray drying [26] reported that polyphenols can react with various components to undergo oxidation and degradation process. References [26,60] evaluated the antioxidant activity of microencapsulated bioactive compounds; they reported that the change in bioactivity was about 10% during a storage period of 6–12 months. However it was reported that TPC values were not altered after a 6 month storage period. It was also found that the type of carrier materials and microencapsulation efficiency affected the stability of the catechin polyphenols.

In this study gum arabic and maltodextrin proved to be more protective against deteriorative agents. The overall results suggest that gum arabic 100% and maltodextrin 100% coating was more beneficial for coating catechin while maltodextrin, gum arabic and chitosan favour retention of TPC and AOC even under harsh conditions and are able to extend the storage shelf-life.

## 3. Experimental Section

### 3.1. Materials

Fresh tea leaves were obtained from Cameron Highlands Malaysia. Maltodextrin (DE 10–16), gum arabic, chitosan were purchased from Scinfield Chemicals (Selangor, Malaysia), and other chemicals were of analytical grade.

### 3.2. Microencapsulation of Green Tea

#### 3.2.1. Preparation of Carbohydrate Microcapsules

To find an appropriate formulation for the feed mixtures of green tea extracts obtained from supercritical fluid technique [68], a simplex centroid mixture experimental design (Table 3) based on previous studies [69] was used. A blend of 40 g (20% *w*/*v*), commercial maltodextrin (MD), gum arabic (GA) and chitosan (CTS) were dispersed in distilled water. Individual wall materials were dissolved individually at 40–60 °C with constant magnetic stirring for 30 min to give a final volumes of 200 mL with a total solids content between 18 and 21 (°Brix). Chitosan was separately dissolved in 5% acetic acid before addition under magnetic stirring and mixed properly. Two grams (5% *w*/*w* based on wall material used) of green tea extract was added to the mixture. The mixture was homogenized using a mini homogenizer (Heidolph-SP-8-Diax900, St. Louis, MO, USA) for 5 min at 8000–9500 rpm until complete dispersion was achieved. The slurry of the water, carrier material and catechin extracts was spray dried in a Buchi 290 mini spray dryer (Buchi, Postfach 9230 Flawil, Switzerland) equipped with a 0.7 mm diameter nozzle at an outlet temperature of 150 ± 5 °C. The inlet temperature was determined by the outlet temperature. The feed flow rate was 15 mL/min. The prepared microparticles were collected using the glass collection chamber. The microparticle powders were placed in aluminium pouches immediately, sealed and kept in a desiccator to cool and to prevent any absorption of moisture until further studies.

#### 3.2.2. HPLC Analysis of Catechins

The five major catechin phenolic compounds present in green tea were analysed by HPLC using the method of Institute for Nutraceutical Advancement for determination of total catechins and gallic acids in green tea (INA Method 111.002) as used by [70] with minor modifications was follows: a reversed-phase C18 column (MetaChem Polaris™ Amide, Agilent Technologies Inc., Santa Clara, CA, USA) C18, 5 µm, 4.6 mm× 250 mm); UV-Vis diode array detector, and a binary solvent system containing acidified water (0.1% orthophosphoric acid-solvent A) and a polar organic solvent (acetonitrile-solvent B) were used. Optimized gradient elution was programmed as follows: 0 min 96% A: 4% B; 12 min 85% A: 15% B; 22 min 75% A: 25% B; 24 min 85% A: 15% B; 30 min 85% A: 15% B and 35 min 96% A: 4% B. The flow rate and injection volume were set at 1 mL/min and 10 µL respectively with a post run of 5 min.The column temperature was 35% while the wavelength was at 280 nm using UV- vis diode array detectors. Five stock solutions of the various catechin standards were prepared by dissolving 1 mg of the individual catechin in 1.0 mL solvent to give a 1000 parts per million (ppm) concentration and stored in the refrigerator at 4 °C. A specified amount of each solution was taken and the five aliquots were mixed and diluted to give a wide range of standard mixtures. The concentration of each catechin in the green tea extract was determined quantitatively based on the chromatographic data of the standard mixture (Figure 2). The catechin components were quantified using the calibration curves of the standards obtained.

#### 3.2.3. Determination Microencapsulation Yield

The method of [71] with some modification was adopted for the determination microencapsulation efficiency. Total catechin of both surface and entrapped core material was determined. A 200 mg sample of encapsulated green tea catechin microparticles was weighed accurately into a test-tube and 2 mL of 50:8:42 (*v*/*v*/*v*) of methanol–acetic acid–water was added. The dispersion was vortexed for 1 min and ultrasonicated for 20 min. The supernatant was centrifuged at 9500 rpm for 10 min and then filtered using a 0.45 µm syringe filter. To determine the surface catechin polyphenols 200 mg of microparticles were treated with a mixture of 2 mL ethanol and methanol (1:1), vortexed for 1 min and was then filtered using a 0.2 µm Millipore syringe filter The amount of total catechin, TPC and DPPH were quantified. The surface bioactive compound (SBC) percentage and the microencapsulation efficiency (ME) of the microparticles were calculated according to the following equations:

SBC = surface bioactive compounds ÷ Theoretical total bioactive × 100
(3)

Microencapsulation efficiency (ME) = 100 − SBC (%)
(4)

#### 3.2.4. Determination of Degree of Swelling of the Microparticles

The methods of [32,48] with some modification were adopted. Pre-weighed catechin microparticles (200 mg) were placed into a previously soaked Visking tubing dialysis bag (2 inf diameter 14.3/26 mm pore size 25 angstroms WMCO 1200–16000 Dalton, ExWorks (London, UK) aseptically placed in a beaker containing 150 mL of simulated gastric and intestinal fluid (pH 2.3 and 7.4, respectively). The content was stirred continuously at 50 rpm and was allowed to swell for 120 min during which the swelling samples were weighed at periodic intervals (0, 20, 40, 60, 80, 100, and 120 min). The percentage degree of swelling of the catechin microparticles was gravimetrically determined using the equation below:

Percentage (%) swelling = (W_t_ − W_O_)/W_O_ × 100 (5)

where W_t_ represents the weight of swollen sample at the stipulated time in the simulated fluid and the initial weight of microparticles before swelling is W_O_.

#### 3.2.5. Investigation of the Release of Encapsulated Catechins

To carry out the in vitro release study the method of [72] with some modification was used. The fluids used for the study included pH 2.3 (0.1 M HCl) and pH 7.4 phosphate buffer as simulated gastric and intestinal fluids (SGF and SIF), respectively. Powdered microparticle samples was suspended in the above solutions (40 mg/mL) and incubated at 37 °C with constant magnetic stirring in generated dialysis tubes (molecular weight cut-off (MWCO) 12000–14000, Spectrum Labs Europe BV, ExWorks. After time intervals 0, 20, 40, 60, 100 and 120 min aliquots from the dialysate simulated solutions were withdrawn in order to determine the TPC, DPPH and total catechin using spectrophotometric and HPLC methods. The result was reported as free catechin that was able to cross the dialysis membrane.

#### 3.2.6. Determination of Moisture Content

The modified method of [36,73] was used in the determination of moisture content of the spray dried microcapsules powders. In brief, 0.20 g samples were weighed in triplicate into a pre-weighed cleaned and dried crucible and dried at 70 °C for between 18–24 h in an oven until a constant weight was obtained. The moisture was calculated using the equation:

M_c_ (%) = W_L_ (g) × 100/W_I_ (g) (6)

where Mc—moisture content, W_I_—weight loss after drying, W_I_—initial weight before drying.

#### 3.2.7. Determination of Water Activity (a^w^)

The water activity was determined using a water activity determination apparatus (Aqua lab CX-2T, Decagon Devices Inc., Pullman, WA, USA). Measurements were carried out in triplicate.

#### 3.2.8. Bulk Density Determination

Bulk density of the green tea microparticles powder was determined using a described method [71]. A 2.0 g sample of the spray dried powder was weighed loosely into a 10 mL measuring cylinder which has been placed on a flat surface. The volume displaced by the powder was recorded. To calculate the bulk density, the weight of the sample was divided by the recorded volume. The tap density was measured by tapping the measuring cylinder containing the microparticles gently for about 3–4 times until a constant volume was obtained. The tap density was calculated by dividing the new volume obtained after the tap by the weight.

#### 3.2.9. Determination of Hygroscopicity (HM) of the Spray-Dried Microparticle Powder

This technique evaluates the capacity of the microparticles to absorb water. The method of [72] was adopted with some modification. Briefly, 0.2 g of green tea powder (encapsulated and free) was weighed and evenly spread on a clean standard Pyrex class Petri dishes (9 cm diameter) after which they were placed in a desiccator at 25 °C and a relative humidity of 74% generated using a saturated brine (NaCl solution). After exposure to these conditions for 120 min the samples were removed from the desiccator and immediately weighed again. The increase in weight was noted. Hygroscopicity was calculated using the formula:

HM % = W_f_ − W_I_/W_I_ × 100 (7)

where W_f_ is final weight, W_I_ is the initial weight.

#### 3.2.10. Determination of Total Phenolics Content (TPC)

The amount of total phenol in the microparticles was estimated colorimetrically following the methods used by the International Organization for Standardization [68,74].

#### 3.2.11. Antioxidant Activity Determination

Antioxidant activity was determined by the catechins’ capacity to scavenge stable DPPH radicals following the method of [74] with slight modifications. Briefly, 200 mg of green tea microparticles were extracted using ethanol (70% *v*/*v*), and 100 µL of the supernatant was added to 3.9 mL of ethanolic solution of 0.1 mM DPPH. The mixture was incubated at room temperature (25 °C) in the dark for 30 min. Sample absorbance (A_S_ sample) was measured using a spectrophotometer (Thermo Fisher Scientific-GENESYS 10S UV.VIS, Waltham, MA, USA) at 517 nm against the absorbance of an ethanol blank (A_B_). Samples were determined in triplicate. The radical scavenging activity was calculated using the formula:

% Inhibition = ((A_B_ − A_S_/A_B_)) × 100 (8)

where A_B_ is the absorbance of the blank and A_S_ is the absorbance of the sample of green tea catechin microparticles.

#### 3.2.12. Storage Stability

The stability of microparticles of catechin was determined by storing 1.5 g in brown vials under different temperature conditions (4 °C, 25 °C, and 40 °C) for 12 weeks, following which 100 mg of the particles were removed and tested for total catechin polyphenols, TPC and DPPH scavenging capacity retained per week using the methods described in Section 2.1.3, Section 2.1.4 and Section 2.4. Samples of tea extracts without encapsulation were used as a control. The percentage catechin polyphenols retained was used for evaluating the storage stability of the green tea extracts in order to predict the shelf-life.

#### 3.2.13. Statistical Analysis

Statistical analysis was carried out using Minitab software version 16 (Pine Hall RD, State College, PA, USA).One way ANOVA was performed to find the significant differences between the samples. Means were compared by using Turkey′s least significant procedure. Significance was defined at the 95% level.

## 4. Conclusions

An accelerated shelf-life study was carried out by exposing spray dried green tea catechin microcapsules and the free (non-encapsulated) grenn teas catechin powder to various storage temperatures. We found out that minimum degradation was observed at lower storage temperature and maximum degradation took place in the free extracts. Catechin compounds were stable irrespective of the carrier materials used. The antioxidant activity decreased during storage at higher temperatures because of degradation. The result indicates that it is better to encapsulate extract powder for increased shelf-life.

## Figures and Tables

**Table 1:** Physicochemical properties of spray-dried green tea microparticles.

Sample Particle	Moisture (%)	Hygroscopicity (%)	Water Activity (a^w^)	Bulk Density (g/cm^3^)	Tap Density (g/cm^3^)	Mean d (4,3) µm
MD:GA:CTS(100:0:0)	3.16 ± 0.06 ^f^	4.55 ± 0.59 ^c^	0.28 ± 0.01 ^h^	0.28 ± 0.04 ^a^	0.35 ± 0.06 ^a^	68.65 ± 5.56 ^c^
MD:GA:CTS(0:100:0)	4.58 ± 0.04 ^c^	4.94 ± 0.34 ^b^	0.26 ± 0.03 ^i^	0.26 ± 0.10 ^b^	0.34 ± 0.08 ^a^	56.68 ± 21.33 ^c^
MD:GA:CTS(75:25:0)	2.49 ± 0.06 ^g^	4.40 ± 0.01 ^cd^	0.31 ± 0.02 ^f^	0.23 ± 0.01 ^ef^	0.34 ± 0.08 ^a^	40.43 ± 3.44 ^c^
MD–GA;CTS (50:50:0)	3.36 ± 0.06 ^e^	4.71 ± 0.03 ^b^	0.34 ± 0.01 ^d^	0.25 ± 0.04 ^bc^	0.33 ± 00 ^ab^	40.93 ± 2.19 ^c^
MD:GA:CTS(25:75:0)	2.31 ± 0.01 ^h^	5.75 ± 0.11 ^ab^	0.34 ± 0.01 ^d^	0.25 ± 0.00 ^bcd^	0.34 ± 0.08 ^a^	43.68 ± 3.20 ^c^
MD:GA:CTS(99:0:1)	4.33 ± 0.05 ^d^	3.22 ± 0.14 ^e^	0.36 ± 0.10 ^b^	0.22 ± 0.003 ^f^	0.29 ± 0.00 ^d^	71.02 ± 1.74 ^c^
MD:GA:CTS(0:99:1)	5.11 ± 0.06 ^ab^	3.94 ± 0.10 ^cde^	0.25 ± 0.05 ^j^	0.24 ± 0.003 ^cde^	0.30 ± 0.06 ^cd^	41.85 ± 9.59 ^c^
MD:GA:CTS(75:24:1)	3.33 ± 0.04 ^ef^	3.46 ± 0.16 ^de^	0.35 ± 0.01 ^c^	0.23 ± 0.00 ^ef^	0.31 ± 0.00 ^bc^	226.79 ± 47.37 ^a^
MD:GA:CTS(50:49:1)	4.32 ± 0.02 ^d^	3.96 ± 0.02 ^cde^	0.32 ± 0.10 ^e^	0.24 ± 0.00 ^d^	0.30 ± 0.06 ^cd^	225.64 ± 25.88 ^b^
MD:GA:CTS(25:74:1)	4.78 ± 0.04 ^b^	3.34 ± 0.04 ^e^	0.34 ± 0.10 ^d^	0.20 ± 0.00 ^g^	0.25 ± 0.04 ^e^	61.98 ± 2.53 ^c^
Crude powder (BLK)	5.45 ± 0.05 ^a^	6.15 ± 0.23 ^a^	0.45 ± 0.01 ^a^	0.16 ± 0.10 ^h^	0.15 ± 0.15 ^f^	nd

Mean that do not share letters vertically are significantly (*p* ≥ 0.05) different. Values are mean of three independent determinations, nd—not determined.

**Table 2:** Regression analysis of degradation reaction rate of green tea catechin in the microparticles prepared with various carrier materials stored at temperatures 40 °C, 25 °C & 4 °C for 12 weeks.

Sample	Parameter	Temperature		
		40 °C	25 °C	4 °C
**1**	Regression equation	y = −0.0143x + 7.550	y = −0.0143x + 7.550	y = −0.0024x + 7.559
	r^2^	0.9503	0.9844	0.9756
	t_1/2_ (weeks)	60	56	288
	ln/k	0.693	0.693	0.693
**2**	Regression equation	y = −0.088x + 7.459	y = −0.138x + 7.449	y = −0.0026x + 7.476
	r^2^	0.9745	0.9193	0.9522
	t_1/2_ (weeks)	38	50	267
	ln/k	0.693	0.693	0.693
**3**	Regression Equation	y = −0.022 + 7.400	y = 0.0219x + 7.424	y= −0.003x + 7.4175
	ln/k	0.693	0.693	0.693
	r^2^	0.9707	0.8837	0.9638
	t_1/2_ (weeks)	36	41	231
	ln/k	0.693	0.693	0.693
**4**	Regression equation	y = −0.0196x + 7.346	y = −0.0154x + 7.314	y = −0.0028x + 3471
	r^2^	0.9925	0.9096	0.9657
	t_1/2_ (weeks)	35	45	248
	ln/k	0.693	0.693	0.693
**5**	Regression equation	y= −0.0305x + 7.681	y = 0.0205x + 7.658	y = −0.0032x + 7.685
	r^2^	0.9858	0.9467	0.9838
	t_1/2_ (weeks)	23	34	217
	ln/k	0.693	0.693	0.693

Samples: 1: MD:GA:CT (0:100:0); 2:MD:GA:CTS (100:0:0); 3: MD:GA:CTS (99:0:1); 4: MD:GA:CTS (25:74:1), 5. MD:GA:CTS (GTE-control), MD—maltodextrin, GA—gum arabic, CTS—chitosan. Values are mean of three independent determinations.

**Table 3:** Experimental design of biopolymer blends of wall material to form microparticles.

Formulation	Maltodextrin (MD, %)	Gum Arabic (GA, %)	Chitosan (CTS, %)
1	100	0	0
2	0	100	0
3	75	25	0
4	50	50	0
5	25	75	0
6	99	0	1
7	0	99	1
8	75	24	1
9	50	49	1
10	25	74	1
